# Barking mad: The vocalisation of the John Dory, *Zeus faber*

**DOI:** 10.1371/journal.pone.0204647

**Published:** 2018-10-03

**Authors:** Craig A. Radford, Rosalyn L. Putland, Allen F. Mensinger

**Affiliations:** 1 Leigh Marine Laboratory, Institute of Marine Science, University of Auckland, Warkworth, New Zealand; 2 Biology Department, University of Minnesota Duluth, Duluth, MN, United States of America; University of Windsor, CANADA

## Abstract

Studies on the behavioural function of sounds are very rare within heterospecific interactions. John Dory (*Zeus faber*) is a solitary, predatory fish that produces sound when captured, but has not been documented to vocalize under natural conditions (i.e. in the wild). The present study provides the first *in-situ* recordings of John Dory vocalisations and correlates them to behavioural response of snapper (*Pagrus auratus*) a common species found through New Zealand. Vocalisations or ‘barks’, ranged between 200–600 Hz, with a peak frequency of 312 ± 10 Hz and averaged 139 ± 4 milliseconds in length. Baited underwater video (BUV) equipped with hydrophones determined that under natural conditions a John Dory vocalization induced an escape response in snapper present, causing them to exit the area opposite to the position of the John Dory. We speculate that the John Dory vocalisation may be used for territorial display towards both conspecifics and heterospecifics, asserting dominance in the area or heightening predatory status.

## Introduction

In taxa as diverse as mammals, birds, reptiles, amphibians, fish, and insects, acoustic signals function in mate choice, resource defence, and species recognition [1, 2]. These signals can provide information about the signallers’ identity, location and condition, thereby reducing the costs associated with direct contact between two competitors. Evidence suggests acoustic signals are often used in mutual assessment during any type of encounter in mammals [3], birds [4, 5], reptiles [6], amphibians [7, 8] and fishes [9]. The vocalisation characteristics may identify the sender, as lower frequency calls usually reflect larger body size in fish [10], or calling rate or sound amplitude may signify fitness (i.e. the body condition of the animal) [3, 11, 12].

Although there are many examples of teleost conspecific vocal interactions, heterospecific agonistic vocalisations are rare [13, 14]. Several species vocalisations are used in mutual assessment and influence the confrontation outcome [9, 15]. For example, the male Lusitanian toadfish (*Halobatrachus didactylus*), may use boatwhistle vocalisations as a warning signal against other males [16]. Also, playback experiments of conspecific sounds in the absence of a resident male have been shown to play a territory guarding role against intrusions from potential competitors in the bicolour damselfish, *Stegastes paritus* [17], and in the painted goby, *Pomatoschistus pictus* [18]. However, studies on the function of sounds in territorial or resource defence are far and few between for heterospecific species, in particular where the vocalizations of known predators induces flight in potential prey.

John Dory, *Zeus faber*, is a solitary predatory fish found worldwide in coastal waters usually less than 200 m deep [19]. They are known to vocalise using a pair of intrinsic sonic muscles, producing a “bark” type sound with a peak frequency of 500 Hz and sound duration of 85 ms [20]. However, to date, there have been no recordings of John Dory vocalisations under natural conditions (i.e. in the wild). Additionally, the effects of the signal displays on other fishes have not been observed. For the first time, the present study details the acoustic and behavioural characteristics of John Dory vocalisations in the wild.

## Materials and methods

This study was conducted in the Cape Rodney to Okakari Point (Leigh) Marine Reserve (549 ha, established in 1976) near Leigh, New Zealand and outside the reserve approximately 4 km due south, near Mathesons Bay. Sampling sites ranged in depth, from 5 to 20 m. Both the marine reserve and adjacent unprotected site are subject to similar environmental conditions and have extensive sub-tidal reef communities typical of moderately exposed coasts in north-eastern New Zealand [21].

Field recordings were taken between January-March 2015, with a hydrophone (ST202, Ocean Instruments Ltd), attached to a baited underwater video (BUV) allowing concurrent behavioural observation. The BUV array consisted of a triangular base of 22 mm diameter stainless steel pipe measuring 1.7 x 1.2 x 1.2 meters with a 1.2 m pole projecting upward from the intersection of the two shortest sides at approximately a 25^o^ angle towards the center of the triangle. Lead weights were added to the base of the triangle to provide stability. A GoPro Hero 4 video camera (GoPro) was attached to the top of the pole facing the base. The camera was encased in the GoPro underwater housing modified with BacPac Backdoor Kit and equipped with a BacPac^TM^ rechargeable battery that allowed recording up to 4 hrs. A SoundTrap 202 recorder (sensitivity 171.8 dB re 1V/μPa; frequency response 20 Hz– 60 kHz) was attached to the pole approximately 0.5 meters from the base and recorded continuously at a sampling frequency of 144 kHz. A 2 l screw top clear plastic bait bottle was affixed with cable ties to the midpoint of the longest side of the base. Two to three 20 cm (Standard Length, SL) pilchard (*Sardinia neopilchardus*) were cross sectioned into approximately 6 equal sections and placed in the jar at the start of each trial for bait.

A total of 21 deployments were undertaken during the hours 08:40 to 14:20, 10 at Cape Rodney and 11 at Matheson’s Bay. For further details about the deployment protocol, see Mensinger et al 2018.

In January 2018, ten John Dory (30–50 cm SL) were captured while recreationally angling (36.2911 S; 174.8184E). After landing, each fish was immediately placed in a 250 litre perspex tank (70 cm long ×60 cm wide × 60 cm height) with a HTI-96-MIN hydrophone (sensitivity -164.4 dB re 1V/μPa; frequency response 2 Hz– 30 kHz; www.hightechincus.com) approximately 30 cm away from the fish’s head to record spontaneous vocalizations. From here on we call these recordings “tank recordings” and the recordings captured with the BUV “field recordings”.

### Ethics statement

John Dory are a recreational fish in New Zealand and current New Zealand rules state that recreational fisherman do not need a licence to catch these fish, there is only a bag limit of 3 fish per boat per day. Therefore, as we were using recreational fishers to catch our fish we only required ethics approval once they handed the fish over for the tank experiments. All tank recording procedures were conducted in accordance with the University of Auckland animal ethics committee (approval # 002001). Special permission to fish in these areas was also not required.

### Analysis

The recordings from each deployment were inspected aurally and visually using Audacity (version 2.0.6). Owing to the presence of other soniferous fish and background noise, manual detection was used, focusing on three distinguishing features: inter-pulse interval, peak frequency and sound duration. Eighty-two vocalisations were subsequently made into 1 second long sound clips. Acoustic analysis was then done using Matlab software (version R2014a). Power spectra were generated for each detected vocalisation using fast Fourier transformation analysis (FFT = 8192 samples) and a Hanning window with 50% overlap before smoothing the result with a triangular window.

Cross correlation and spectral coherence analysis was used to verify the field recordings to tank recordings of John Dory. The frequency range, peak frequency, sound duration, number of pulses per call, pulse duration and inter-pulse interval of each John Dory vocalisation were calculated and averaged. The vocalisations captured from the tank recordings were subjected to the same analysis as the field recordings. These results were then tested for significant differences using an unbalanced Mann-Whitney Rank Sum Test because the datasets failed normality (Shapiro-Wilk, P < 0.05).

All video recording was conducted with the Go Pro camera (fish lens option) with a recording rate of 29.97 frames per second. The viewing field of the camera was approximately 2.25 m^2^, which included most of the triangular base, the bait container and 1 m^2^ area outside the triangle. The John Dory vocalization was detected in the audio track of the video record. Frame by frame analysis with Photoshop Software determined the orientation of the snapper just prior to vocalization. A line was drawn through the longitudinal axis of the fish to determine its orientation, with the top of the video frame considered 0^o^. Most sounds evoked startle response or C-start, because of the shape of the body following contraction of the trunk muscles on one side of the body [22, 23] The orientation of the fish 100 ms prior to the c-start and the orientation of the fish as it exited the frame were determined. Pre- and post-sound orientations were analysed using the Watson-Williams F-test in Oriana (version 4; Kovach Computing Systems; Wales, UK) with Rao’s Spacing Test to determine if the angular data was randomly distributed and whether the orientations were significantly different.

## Results

### Acoustic characteristics

**Fig 1:**
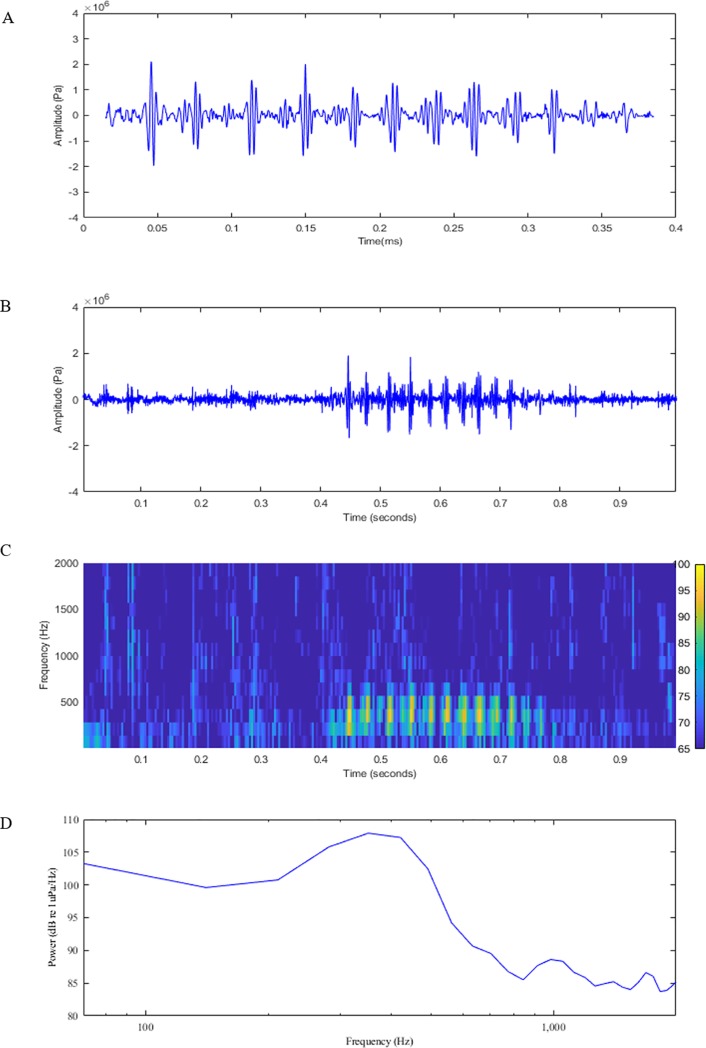
Acoustic visualisations of the John Dory bark. (A) zoomed waveform showing multiple pulses; (B) waveform showing multiple pulses; (C) spectrogram showing the frequency bandwidth; and (D) spectra showing the peak frequency.

**Fig 2:**
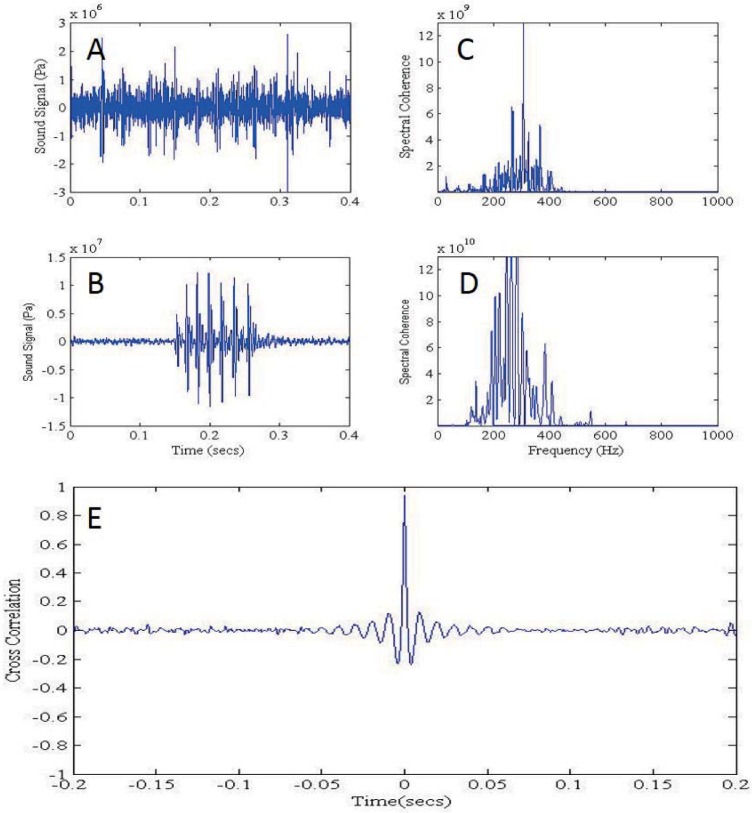
Examples of John Dory vocalisation waveforms of tank (A) and field (B) recorded fish. Examples of the spectral coherence between the John Dory vocalisations recorded in the tank (C) and the field (D). Example of cross correlation analysis of the field and tank recorded John Dory vocalisations (E).

Out of 21 video/hydrophone deployments 82 John Dory vocalisations and associated fish behaviours were recorded. In addition, 10 tank vocalisations from 10 John Dory were recorded. John Dory vocalisations consisted of short repeated low frequency pulses (Fig 1A), which were termed by Onuki & Somiya [20], who recorded the sound in air, as “barks”. To confirm sounds were produced by John Dory the spectral content (Fig 2A–2D) and cross correlation (Fig 2E) of the field recordings were compared to the tank recordings. The peaks in the spectra between the two recordings matched closely and the cross correlations which ranged from 0.75 to 0.99, also suggested they matched.

The frequency range of the John Dory bark ranged from 200–500 Hz in the field recordings compared to the tank recordings where the frequency range was wider, 200–600 Hz (Table 1; Fig 1). The mean duration of the field recorded vocalisations was 197 ± 8 ms with a peak frequency of 312 ± 10 Hz compared to tank recorded John Dory vocalisations, which were significantly shorter (139 ± 4 ms; U = 217.5, P < 0.05) and had a significantly higher peak frequency (414 ± 20 Hz; U = 145.0, P < 0.05). Analysing the number of pulses per vocalisation, there were 6 ± 0 pulses per call in field recordings and a similar number at 8 ± 0 (U = 1.1, P > 0.05) from the tank recordings, with mean pulse duration of 22 ± 1 ms and 19 ± 1 ms, respectively. However the inter-pulse interval (U = 180.1, P < 0.05) and pulse period (U = 120.2, P < 0.05) was significantly longer for vocalisations produced in the field (3 ± 0 ms; 25 ± 1 ms, respectively) than in the tank (2 ± 1 ms, 20 ± 1 ms, respectively).

**Table 1:** Acoustic characteristics of the John Dory vocalisation comparing field and tank recorded fish.

	Field Recordings	Tank Recordings
Frequency Range (Hz)	200–500	200–600
Peak Frequency (Hz)	312 ± 10	414 ± 20 *
Sound Duration (ms)	197 ± 8	139 ± 4 *
Number of Pulses	6 ± 0	8 ± 0
Pulse Duration (ms)	22 ± 1	18 ± 1
Inter-Pulse Interval (ms)	3 ± 0	2 ± 0 *
Pulse Period (ms)	25 ± 1	20 ± 1 *

* signifies significant differences between water and air recordings (P < 0.05).

### Behaviour

**Fig 3:**
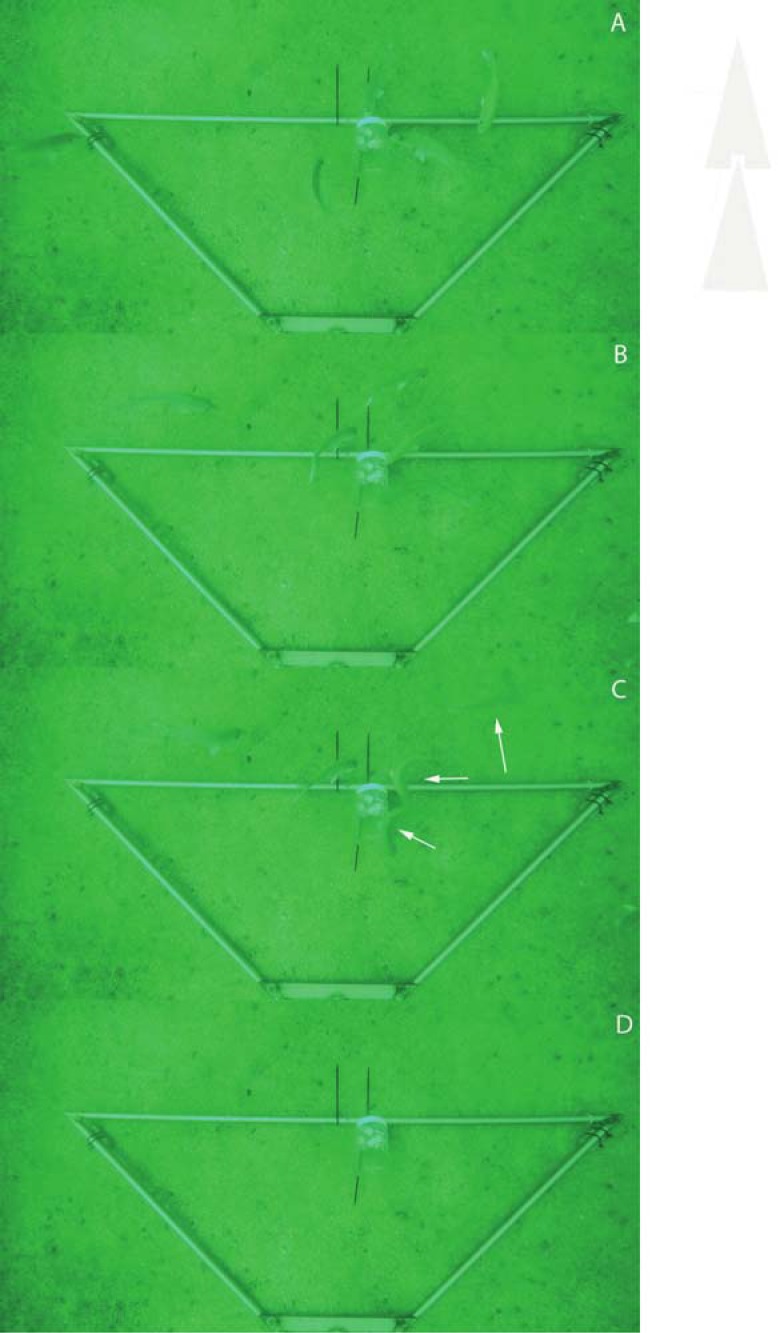
A series of stills from the Go Pro video’s showing an example of what was observed. (A) Australian snapper (*Pagus auratus*) coming into the field of view after being attracted by the bait pot; (B) numbers of Australian snapper increase over time; (C) a John Dory vocalisation is heard by the Australian snapper and they exhibit the traditional c-start response (white arrows); and (D) the field of view is empty within seconds of the John Dory vocalisations. Scale: the horizontal side of the triangular base is 1.7m.

**Fig 4:**
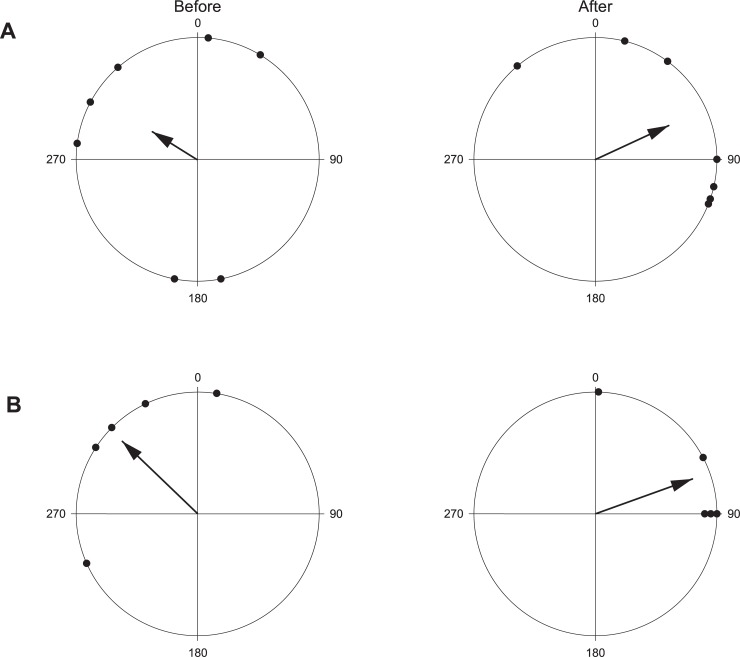
Polar plots showing the anterior/posterior orientation at the BUV (A) just prior to John Dory sound and (B) as they swam out of the camera range following John Dory sound. In both instances John Dory entered the frame from approximately 200°. Orientations for A and B were significantly different before and after [Watson-Williams F-test (A- P = 0.022; B-P = 0.003)].

**Fig 5:**
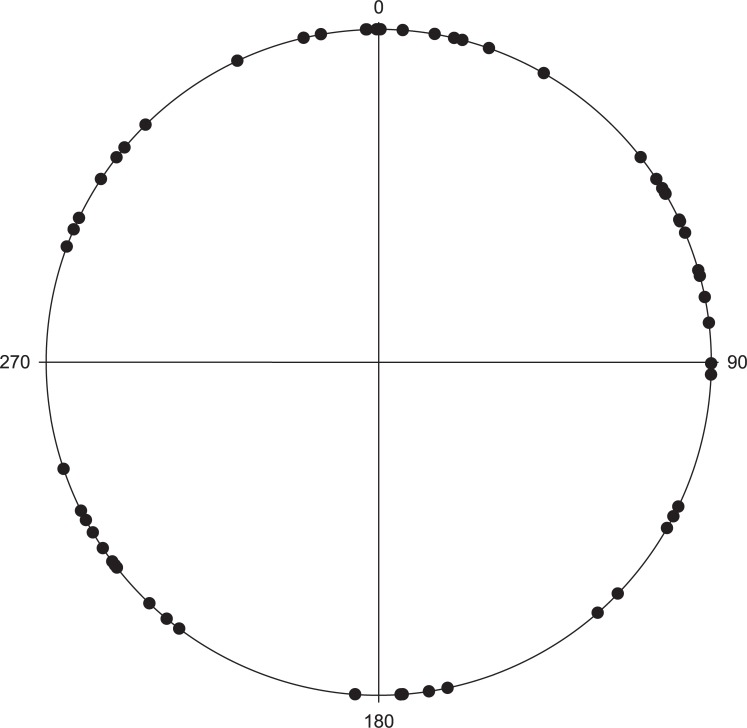
Polar plots showing the distribution of snapper leaving video frame during a 5-minute period at MBD (N = 55) when no John Dory sound. Angles are relative to the bait jar lid and 270° is representative of the vertical pole frame.

Video recordings (see supplementary information) showed that John Dory vocalisations immediately elicited a c-start response in snapper (Fig 3). The mean orientation of snapper changed considerably pre and post John Dory vocalisation (Fig 4A, the mean vector was 315.3^o^ vs 69.4^o^ and in Fig 5B, it was 303.2^o^ vs 68.2^o^, respectively; John Dory entered the camera frame at approximately 200^o^ in both instances). The orientation angles were found to be significantly different (A- P = 0.022; B-P = 0.003) between the before and after orientations. In the absence of John Dory sound, the direction Australasian snapper left the video frame, was uniformly distributed during a 5-minute sampling (P = 0.419, Fig 5).

## Discussion

John Dory is a solitary predator known to produce vocalisations using a pair of intrinsic sonic muscles, but to date these vocalisations have only been recorded in air [20]. The present study is the first record of the acoustic and behavioural characteristics of John Dory vocalisations in the wild. The typical ‘bark’ recorded underwater in this study had an average duration of 197 ± 8 ms (n = 82), significantly longer compared to the duration of the tank recorded fish, 139 ± 4 ms (n = 10) as well as those stated by Onuki and Somiya [20], 87 ms ± 10 sd (n = 50). Additionally, the peak frequency of the field recorded fish were significantly lower at 312.9 ± 10.8 Hz compared to the tank recordings (414.8 ± 20.5 Hz) and approximately 370 Hz for Onuki and Somiya [20] recordings.

Comparing animal vocalisations in the field recordings to tank recordings is difficult due to the possibility of the tank altering the acoustic structure of the call or inducing changes in the vocal behaviour (see Pavuliscu [24] and Akamatsu et al. [25]). Here, field and tank recordings of John Dory vocalisations show various degrees of differences (Table 1) between number of pulses within the call and most importantly the peak frequency. Therefore, it is imperative comparisons of fish vocalisations recorded in small tanks are made with caution and that the best assessment are fish vocalisations recorded in its natural habitat.

The use of concurrent BUV during our experiment provided evidence that after a John Dory vocalised, Australasian snapper (*Pagrus auratus*), exhibited a classic startle response (c-start), moving suddenly away from the bait source. The functional significance of the vocalizations are unknown. One potential explanation could be territorial display [13]. Occasionally two John Dory were visible in the video after a vocalisation, therefore vocalisation could be a territorial display or a social interaction with conspecifics. Additional data of John Dory vocalising in groups of two or more would confirm this hypothesis, and it would be prudent to know the sex of individuals during group interactions. However, there did not appear to be any change in the calling parameters during a single deployment, suggesting that only one John Dory was vocalising. Interestingly, all recordings taken by Onuki and Somiya [20] were from female John Dory, however males may also have the capacity to be vocal as the same paper showed no sexual dimorphism in sonic muscle size.

There have been numerous studies showing how conspecific sounds can be used for territory and resource defence, where the outcome of the encounter is dictated by the receivers being able to recognise the size of their opponent from the call. The BUV was very successful at attracting and maintaining large groups (10+) of snapper in the area [26]. These fish readily attacked the bait jar despite the bait not being readily accessible during the 30 min soaks. However, it was unlikely that the calls were to remove competition for this resource as the John Dory never attempted to interact with the bait jar. For example, in the croaking gouramis, *Trichopsis vittata*, croaking during agonistic displays enhance the probability of winning the encounter and attaining dominance [27]. In this species, the acoustic features related to resource defence was body size, which was characterised by the sound pressure level and dominant frequency of the vocalisation [28].

However the clear startle response observed could be an indication of territorial display between heterospecifics, with the John Dory asserting its dominance over other fish habiting the area such as the Australasian snapper. It could also be associated with a secondary predator entering the vicinity and the snapper have co-evolved to recognize the John Dory call as a warning.

In conclusion, this is the first record of John Dory vocalisations and associated behaviours in their natural environment. John Dory is a predatory fish, therefore the observations observed here are at odds with this; that is why would a predatory fish produce a sound to alert potential prey they were around? Resource defence and feeding strategy are unlikely to explain the vocalisation, therefore we speculate the sound is used as a territorial display of asserting its presence, both for conspecific and heterospecifics alike.
